# Health Tract, No. 144

**Published:** 1865

**Authors:** 


HEALTH TRACT, No. 144.

### "Catching Cold."

A LARGE number of fatal diseases result from taking cold, and often from such slight causes, apparently, as to appear incredible to many. But, although the causes are various, the result is the same, and arises from the violation of a single principle, to wit, cooling off too soon after exercise. Perhaps this may be more practically instructive if individual instances are named, which, in the opinion of those subsequently seeking advice in the various stages of consumption, were the causes of the great misfortune, premising that when a cold is once taken, marvelously slight causes serve to increase it for the first few days—causes which, under ordinary circumstances, even a moderately healthful system would have easily warded off.

Rachel, the tragedienne, increased the cold which ended her life, by insufficient clothing in the cars, in traveling from New-York to Boston; such was her own statement.

The immediate cause of the last illness of Abbott Lawrence, the financier and the philanthropist, was an injudicious change of clothing.

An eminent clergyman got into a cold bed in mid-winter, within fifteen minutes after preaching an earnest discourse; he was instantly chilled, and died within forty-eight hours.

A promising young teacher walked two miles for exercise, and on returning to his room, it being considered too late to light a fire, sat for half an hour reading a book, and before he knew it a chill passed over him. The next day he had spitting of blood, which was the beginning of the end.

A mother sat sewing for her children to a late hour in the night, and noticing that the fire had gone out, she concluded to retire to bed at once; but thinking that she could "finish" in a few minutes, she forgot the passing time, until an hour more had passed, and she found herself "thoroughly chilled," and a month's illness followed to pay for that one hour.

A little cold taken after a public speech in Chicago, so "little" that no attention was paid to it for several days, culminated in the fatal illness of Stephen A. Douglas. It was a slight cold taken in midsummer, resulting in congestion of the lungs, that hurried Elizabeth Barrett Browning to the grave within a week. A vigorous young man laid down on an ice-chest on a warm summer's day, fell asleep, waked up in a chill, which ended in confirmed consumption, of which he died three years later. A man in robust health and in the prime of life began the practice of a cold bath every morning, getting out of bed and standing with his bare feet on a zinc floor during the whole operation; his health soon declined, and ultimately his constitution was entirely undermined.

Many a cold, cough, and consumption are excited into action by pulling off the hat or overcoat as to men, and the bonnet and shawl as to women, immediately on entering the house in winter, after a walk. An interval of at least five or ten minutes should be allowed, for however warm or "close" the apartment may appear on first entering, it will seem much less so at the end of five minutes, if the outer garments remain as they were before entering. Any one who judiciously uses this observation, will find a multifold reward in the course of a lifetime.

